# Sex and Age Differences in Myocardial Fibrosis: Do Sex Hormones Address the Knowledge Gap?∗

**DOI:** 10.1016/j.jacadv.2023.100332

**Published:** 2023-05-10

**Authors:** Emily S. Lau, Gurleen Kaur, Garima Sharma

**Affiliations:** a Division of Cardiology, Massachusetts General Hospital, Boston, Massachusetts, USA; b Department of Medicine, Brigham and Women’s Hospital, Boston, Massachusetts, USA; c Division of Cardiology, Johns Hopkins University School of Medicine, Baltimore, Maryland, USA

**Keywords:** myocardial fibrosis, sex hormones, women

**Figure:**
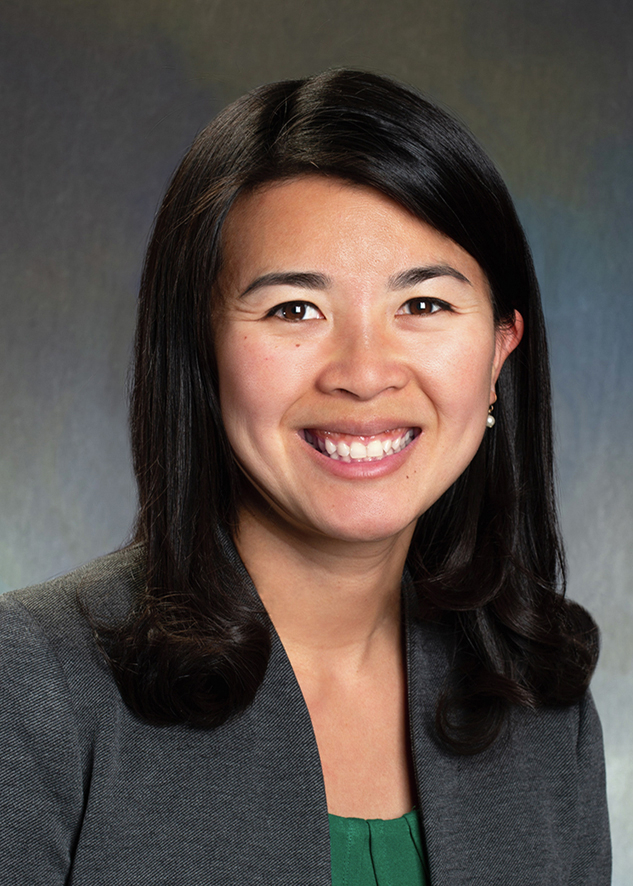

“We have studies of fruit flies, mice, hamsters, frogs, monkeys, and men with this condition—but medical research using women as subjects just never occurred to anybody.”^1^

Since Etta Hulme first satirized the historical underrepresentation of women in medical research in the September 1991 publication of the *Fort Worth Star Telegram*, sexual dimorphism in cardiovascular disease (CVD)—from disease susceptibility, pathogenesis, presentation, and response to treatment—has become established knowledge.^2^ For example, despite a similar lifetime risk of developing heart failure (HF) in both men and women, the prevalence of HF with preserved ejection fraction is markedly higher in women vs men.^3^ Still, the mechanistic underpinnings of these sex-based differences remain elusive. Recent studies suggest that sex-specific patterns of age-related cardiac remodeling, specifically fibrotic cardiac remodeling, may offer mechanistic insights into the differences in CVD phenotype in men vs women.^4^ Fibrotic cardiac remodeling is a pattern of cardiac aging that may explain the increased predilection to left ventricular (LV) hypertrophy and diastolic dysfunction with older age,^5^^,^^6^ and sex-based differences may explain why women more likely develop concentric remodeling and accentuated diastolic dysfunction with impaired LV relaxation in response to age and pressure overload while men develop eccentric and concentric remodeling.^7^ Cardiac fibrosis can be classified into 2 patterns: replacement and interstitial fibrosis. Replacement fibrosis is characterized by replacement of damaged cardiomyocytes with collagen-containing scar that can be seen after direct cardiac injury like in the case of myocardial infarction.^8^ Less recognized is interstitial fibrosis which is characterized by predominant extracellular matrix deposition without loss of cardiomyocytes that can occur in conditions like hypertensive heart disease, aortic stenosis, and nonischemic dilated cardiomyopathy.^9^ The widespread adoption of cardiac magnetic resonance (CMR) imaging has enabled noninvasive evaluation of both patterns of fibrosis. Specifically, late gadolinium enhancement (LGE) allows for assessment of replacement myocardial fibrosis, while T1 myocardial mapping and extracellular volume (ECV) help quantify myocardial extracellular expansion and interstitial fibrosis.^10^

Notable sex differences in cardiac fibrosis have been observed. In a prior analysis of the MESA (Multi-Ethnic Study of Atherosclerosis), CMR evaluation revealed that women had higher pre-contrast T1 times and ECV and lower post-contrast T1 times compared with men, consistent with greater diffuse myocardial fibrosis. Furthermore, age was significantly associated with all T1 indices (marker of diffuse myocardial fibrosis) in men, while age was only correlated with specific indices (gadolinium clearance rate and ECV) in women, highlighting differences in age-related extracellular cardiac remodeling in men vs women.^11^ Could sex hormones explain why age-related cardiac remodeling, particularly interstitial fibrosis, differs so dramatically in men and women? After all, sex hormone levels change considerably with age. Men experience a steady decline in testosterone after age 30, while the transition of menopause is accompanied by a dramatic shift in the hormonal milieu in women, characterized predominately by a decline in estrogen.^12^^,^^13^ Testosterone levels also fluctuate in women, with gradual decrease across early reproductive years followed by an increase after the onset of menopause.^14^ Few clinical and population-based studies have examined the effect of sex hormones on cardiac remodeling and fibrosis, highlighting an important unmet research need (Figure 1).

**Figure 1:**
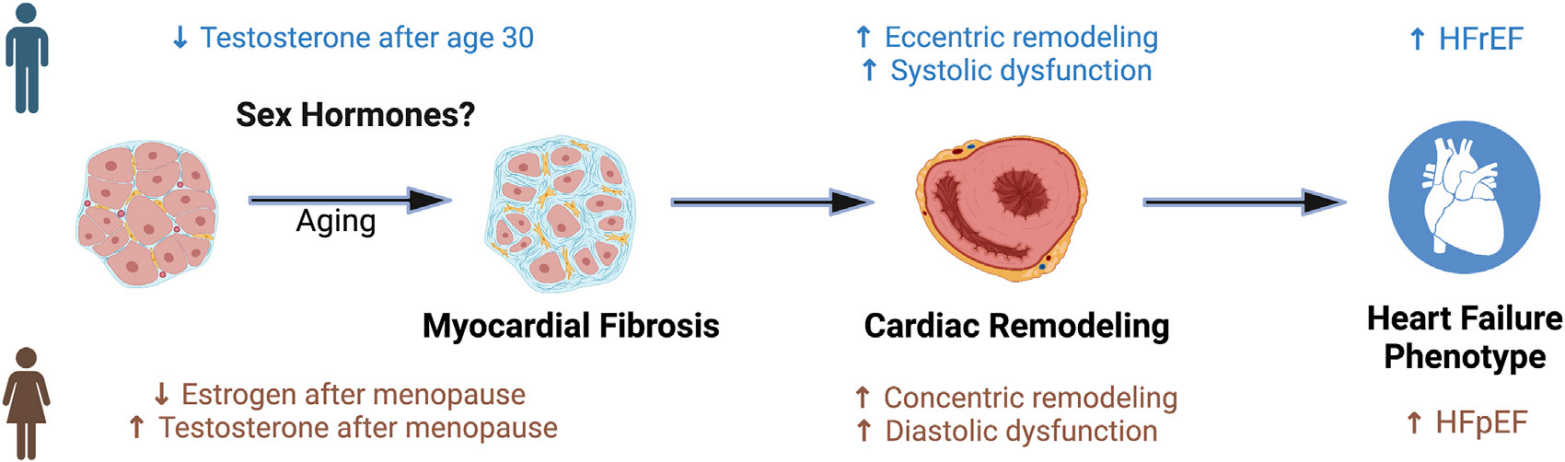
**Sex Differences in Age-Related Cardiac Remodeling May Explain Phenotypic Differences in Heart Failure With Sex Hormones Playing a Potential Role in the Process** HFpEF = heart failure with preserved ejection fraction; HFrEF = heart failure with reduced ejection fraction.

In this issue of *JACC: Advances*, Chehab et al^15^ explore the relationship between endogenous sex hormone levels and markers of interstitial myocardial fibrosis (assessed by ECV and native T1 on CMR) and replacement myocardial fibrosis (assessed by LGE) in men and postmenopausal women. The authors included 2,324 patients from MESA that had sex hormones measured at baseline and CMR 10 years later. Analyses were stratified by sex and age (45-54, 55-64, ≥65 years) and adjusted for clinical covariates and other CMR-related measurements including LV function, LV mass index, and LV end-diastolic volume index. Women were older and had higher LV end-systolic volume, LV end-diastolic volume, and LV ejection fraction, while men had greater LV mass.^15^ Analysis of baseline endogenous sex hormones revealed decreasing levels of free testosterone and increasing levels of sex hormone-binding globulin (SHBG) with advancing age in men and decline in estradiol and increase in SHBG with age in women. With respect to CMR indices, men experienced an increase in T1 and ECV, consistent with the development of interstitial fibrosis, while women developed lower T1 and experienced no difference in ECV, consistent with no change in interstitial fibrosis with age.^15^

Exploration of the relationship between endogenous sex hormones and markers of interstitial myocardial fibrosis found notable associations between testosterone and SHBG with interstitial fibrosis in older men. In multivariable linear regression analyses, there was a significant association of free testosterone, bioavailable testosterone, and SHBG with ECV and T1 in men >age 65 years only. Specifically, a 1-standard deviation increase in log-transformed free testosterone was associated with 2.45% lower ECV (β = −2.45, *P* = 0.02) and 21.5% lower native T1 (β = −21.5, *P* = 0.03). There were no significant associations noted in other age groups. By contrast, there was a significant association of total testosterone with lower native T1 in women in the 55 to 64 age group only. No significant association was noted in other age groups or for total testosterone and ECV in postmenopausal women.^15^

Evaluation of sex hormones and replacement fibrosis was conducted in a subset of 943 men with available LGE CMR data for assessment of myocardial scar and replacement myocardial fibrosis. Findings were again limited to men older than age 65 with higher levels of estradiol associated with greater risk of myocardial scar (OR: 4.10; 95% CI: 1.35-12.40; *P* = 0.01).^15^

This work represents the first population-based study in a large multiethnic diverse population investigating the relationship between endogenous sex hormones with interstitial and replacement myocardial fibrosis. While prior studies have demonstrated that higher testosterone levels are associated with lower risk of CVD in men^16^ and greater risk of CVD in women,^17^ the relationship between testosterone and myocardial fibrosis has not been well studied. In a prior population-based cross-sectional analysis, men with LV hypertrophy had lower levels of total testosterone, but the correlation was no longer significant after adjusting for body mass index.^18^ In another prospective cohort study, low levels of testosterone were independently associated with greater risk of incident HF in men.^19^ Chehab et al^15^ show that higher testosterone is associated with lower interstitial myocardial fibrosis and higher estradiol is associated with greater odds of myocardial scar as assessed by LGE in older men (age >65 years). Why the relationship between sex hormones with both interstitial and replacement myocardial fibrosis was restricted to older men is unclear and warrants further study. Previous mouse studies have shown that estradiol treatment impairs contractile function in male cardiomyocytes.^20^ The authors propose that higher estrogen may contribute to atherosclerotic CVD development in men, leading to myocardial infarction and subsequent myocardial scar.^13^

Separately, Chehab et al^15^ describe a significant association between higher testosterone and lower interstitial fibrosis in postmenopausal women, but only in the 55 to 64 years age group. This differs from previous studies that found that a more androgenic profile was associated with greater LV mass in postmenopausal women.^17^ The biological plausibility of significant correlation in only one specific age group is unclear.

Chehab et al^15^ nicely highlight the potential relationship between sex hormones and fibrosis, but their results should be cautiously interpreted given several limitations. The cross-sectional design does not allow for the establishment of a causal relationship. In addition, analyses were not adjusted for multiple hypotheses testing so significant results may reflect statistical chance alone, and thus findings are solely exploratory. Overall, this important work by the authors suggests that sex hormones are linked to myocardial fibrosis, but further research is warranted to further substantiate their findings and to ultimately, illuminate ways to modify hormone levels for risk reduction and clinical benefit.

## Funding support and author disclosures

Dr Lau has received modest honoraria from Roche Diagnostics; and is supported by the 10.13039/100000968American Heart Association (853922) and the 10.13039/100000002National Institutes of Health (K23-HL159243). Dr Sharma is supported by the 10.13039/100000968American Heart Association (979462), unrelated to this work. All other authors have reported that they have no relationships relevant to the contents of this paper to disclose.
